# Motivators to participation in HIV vaccine trials and cancer trials: the application of personal and social categorization

**DOI:** 10.1186/1742-4690-9-S2-P110

**Published:** 2012-09-13

**Authors:** S Dhalla, G Poole

**Affiliations:** 1 University of British Columbia, Vancouver, Canada; 2 School of Population and Public Health, University of British Columbia, Vancouver, Canada

## Background

The Health Belief Model provides a framework to understand motivators for volunteering for medical research. Motivators can take the form of social benefits, including macrosocial (pertaining to one’s greater society), mesosocial (pertaining to one’s larger social world), and microsocial (pertaining to one’s immediate social network). Personal benefits can be sub-divided into psychological, physical, and financial well-being. In this systematic review of review articles, we extend our conceptual framework from motivators regarding participation in HIV vaccine trials to another life-threatening disease, cancer.

## Methods

In 2012, two people independently searched the Cochrane Database for Systematic Reviews, Pubmed, Embase, and Google Scholar to identify review articles examining cancer trial motivators to participation. Search terms were: “cancer”, “oncology”, “cancer trials”, “oncology trials”, “clinical trials”, “medical research”, “willingness to participate”, “motivators”, “incentives”. Review articles were also retrieved from our search examining barriers to participation in cancer research and from bibliographic references.

## Results

We retrieved 12 review articles from 2000-2012 examining motivators to participation in cancer trials. Personal benefits were most often psychological such as “coping with symptoms”. Social benefits included “advancing research”, “helping other cancer patients”, and “for their family”. These categories also apply well in HIV vaccine trials. Personal/psychological benefits are most commonly cited as motivators for both types of research. In cancer research, “coping with symptoms” and “extending life expectancy” were motivators; in HIV vaccine trials, it was to “reduce the risk of becoming HIV positive”.

## Conclusion

While specific motivators vary between considerations of cancer research and HIV vaccine trials, these motivators fall into similar categories at similar frequencies. Personal / psychological benefits are most common in each. Altruistic factors found at a macrosocial level outnumber factors relevant to more proximal levels, such as family and other patients. Participant recruitment must be mindful of these categories of motivators for both HIV vaccine and cancer research.

